# LOMDP: Maximizing Desired Opinions in Social Networks by Considering User Expression Intentions

**DOI:** 10.3390/e27040360

**Published:** 2025-03-29

**Authors:** Xuan Wang, Bin Wu, Tong Wu

**Affiliations:** School of Cyberspace Security, Beijing University of Posts and Telecommunications, 10 Xitucheng Road, Haidian District, Beijing 100876, China; buptwx@bupt.edu.cn (X.W.);

**Keywords:** positive opinion maximization, opinion dynamics, entropy, information theory, desired opinion maximization

## Abstract

To address the problem of maximizing desired opinions in social networks, we present the Limited Opinion Maximization with Dynamic Propagation Optimization framework, which is grounded in information entropy theory. Innovatively, we introduce the concept of node expression capacity, which quantifies the uncertainty of users’ expression intentions via entropy and effectively identifies the impact of silent nodes on the propagation process. Based on this, in terms of seed node selection, we develop the Limited Opinion Maximization algorithm for multi-stage seed selection, which dynamically optimizes the seed distribution among communities through a multi-stage seeding approach. In terms of node opinion changes, we establish the LODP dynamic opinion propagation model, reconstructing the node opinion update mechanism and explicitly modeling the entropy-increasing effect of silent nodes on the information propagation path. The experimental results on four datasets show that LOMDP outperforms six baseline algorithms. Our research effectively resolves the problem of maximizing desired opinions and offers insights into the dynamics of information propagation in social networks from the perspective of entropy and information theory.

## 1. Introduction

With the rapid development of information technology, social networks (e.g., Twitter and Facebook) are widely used and have become leading platforms for people to express and exchange their opinions. The propagation of opinions on social media has profoundly affected many fields, such as business, finance, and the military. Analyzed through the lens of information theory, opinion guidance can even cause fluctuations in the stock market and have a significant impact on social stability. In marketing, the spread of desired opinions about products and stores can create a good reputation, which can result in significant economic benefits. Therefore, the Influence Maximization (IM) [1] problem has been widely studied to identify the most influential nodes and accelerate information spread in a social network. However, the IM problem has many limitations in practical applications, where people may hold both desired and undesired opinions about an item. Thus, even if a product is widely recognized, its poor reputation can still hinder it from achieving the anticipated impact. The importance of research on the positive opinion maximization (POM) problem [2] is beginning to come to the forefront.

The positive opinion maximization problem focuses on maximizing the spread of “desired” opinions—whether positive, negative, or neutral—by activating specific seed nodes to facilitate their dissemination. The type of opinion designated as the “desired” one can vary, as it is up to the opinion propagator to determine which opinions to prioritize. However, regardless of which opinion is chosen, the underlying algorithm remains the same. This flexibility has made the problem an area of significant interest, given its potential to bring about positive social and economic outcomes.

Research on the desired opinion maximization issue is very challenging, as both desired and undesired opinions can spread simultaneously in discussions on controversial topics, political affiliations, and merchandising. Related to disease transmission issues [3], unlike in opinion propagation, it is difficult to block the spread of negative opinions by controlling negative individuals, and in the selection of seed nodes, the influence factor of a node cannot be the only consideration. For example, if a publisher promotes a ball game, they will rarely choose to invite a famous music master; instead, they will prefer a sports superstar to promote it. Moreover, people will not be interested in all opinions, and there will inevitably be a silent crowd for specific opinions. Meanwhile, when determining the seed node, the objective function has been shown to no longer satisfy monotonicity and submodularity [4], and the propagation of desired opinions cannot be expanded simply by increasing the number of seeds. From an entropy perspective, during the opinion propagation process, the opinions of nodes are not static but change dynamically over time and with the opinions of their neighbors. All these factors present challenges in solving the desired opinion maximization problem.

Based on the above discussion, there are still several shortcomings in the current research on the DOM problem, specifically the following challenges: (i) Current research does not take into account the potential existence of silent nodes in the propagation of opinions, nor does it address the changes in seed selection and opinion dynamics in the presence of silent nodes. In practical scenarios, some users may choose to remain silent due to their individual personalities, the diminishing interest in the event, or social pressures, all of which can influence the propagation of opinions [5]. (ii) The positive benefits of nodes are difficult to determine accurately, and the existing seeding method often involves a one-time selection, which may lead to the inefficient use of resources. (iii) The combination of activation nodes and the dynamic opinion formation process is neglected, and the impact of silent nodes on the dynamic evolution of opinions is not adequately considered. (iv) Activation nodes can serve as the basis for the development of a new opinion formation process, while silent nodes are not considered in this dynamic opinion formation process.

To address the challenges outlined above, this paper proposes a novel algorithm for maximizing the spread of desired opinions within signed social networks. The methods employed in our approach are grounded in the POM framework, which focuses on maximizing the dissemination of the opinions that the information disseminator seeks to spread. The main contributions of this paper are summarized as follows:

(1) We propose the concept of expression capacity, which determines whether a user becomes a silent node at a given moment based on factors such as user personality, event heat, and group effects. To the best of our knowledge, this is the first time that the influence of silent nodes has been introduced into the desired opinion maximization problem, and it is the first time that the decay factor of event heat has been taken into account in the dynamics of opinion propagation.
(2) To address the seed selection limitations in previous algorithms, we propose the Limited Opinion Maximization (LOM) algorithm, which divides networks into communities, evenly places seeds, and activates nodes with maximal expected returns in multiple stages, thereby significantly enhancing propagation effectiveness.
(3) We propose the Limited Opinion Dynamic Propagation Optimization Model (LODP), an improved dynamic opinion model that integrates the classical FJ model with finite confidence opinions and silent node influence, thereby substantially enhancing the accuracy of dynamic opinion modeling.
(4) We evaluate the effectiveness of the proposed method by comparing it with several baseline algorithms using four social network datasets, demonstrating its superior performance.

These contributions advance opinion dynamics theory under entropy constraints while delivering real-world solutions. For instance, in public health, the LOMDP framework identifies influential non-silent nodes to design targeted vaccination campaigns. In crisis management, its modeling of silent user behavior enables entropy-mitigated communication strategies, demonstrating cross-domain applicability in sectors that require opinion guidance.

The rest of the paper is organized as follows: Section 2 reviews related work, Section 3 describes the framework and algorithms of the model, Section 4 outlines the design and results of the experiments, and Section 5 provides the concluding remarks on the experiments.

## 2. Related Work

### 2.1. Influence Maximization

The Opinion Maximization problem originated from the study of the Influence Maximization (IM) problem. Domingos and Richardson et al. [6] were the first to propose the IM problem and study it. If the selection cost of the node u is $C_{s}\left(u\right)$ and the total budget is B, then the aim of the IM problem is to select k seed nodes, S, in a social network, $G=\left(V,E\right)$, such that the final propagation impact is maximized, i.e., $S\leftarrow {argmax}_{S^{*}\subseteq V\wedge \left|S^{*}\right|=k\wedge c_{s}\left(S^{*}\right)\leq B}\sigma \left(S^{*}\right)$ [7]. Current research on the IM problem can be broadly categorized into two directions: greedy and heuristic-based algorithms.

1. Greedy algorithm

Kempe et al. [8] proposed that the IM problem can be solved using a greedy algorithm, where the node with the largest increment in set influence is selected as the new seed node each time until the budgeted value is reached, a process that requires extensive Monte Carlo simulation [8]. However, the time complexity is too high. To improve the efficiency of the algorithm, Leskovec et al. [9] proposed the CELF algorithm to optimize the calculation of node influence by using the submodular property of the objective function of the IM problem. The speed is nearly 700 times higher than that of the greedy algorithm under the same influence effect. On this basis, Goyal et al. [10] optimized the CELF algorithm using the submodular properties of the propagation function and proposed the CELF++ algorithm, which circumvents the marginal effect of the CELF algorithm, reduces a large number of repeated calculations, and runs 35–55% faster than the CELF algorithm [11]. In recent years, there have been many advancements in the improvement of the greedy algorithm. For example, Azaouzi et al. [12] proposed a SAIM model based on the greedy algorithm, which uses the PageRank algorithm [13] to calculate users’ influence values in social networks. Additionally, it employs the “influence BFS tree” model to identify a set of nodes with the most influential nodes. The SAIM model effectively computes influence while also considering the variability of user opinions. However, in the POM problem, the objective function does not satisfy monotonicity or sub-modeling; thus, the results obtained using the greedy approach often do not achieve the desired effect, the computational complexity is high, and the overhead is significant in large networks.

2. Heuristic algorithm

Basic heuristics include various centrality algorithms, such as degree centrality [14], proximity centrality, mediator centrality, and eigenvector centrality [15]. All of these algorithms calculate the influence of nodes based on certain rules, but they are based on a single metric to calculate the centrality of nodes, and they do not take into account the global characteristics of the network or the attributes of the nodes. In this regard, Wang et al. [16] proposed the Degree Discount algorithm to select seed nodes by lowering the priority of highly connected nodes, which improves selection efficiency and calculation accuracy; however, this approach is only based on static network and local information, and it does not take into account the dynamic changes in the network. Chen et al. [17] proposed the MIA algorithm to optimize the information dissemination strategy by maximizing the sphere of influence and identifying key nodes, but its computational complexity is high and unsuitable for dealing with large networks. Borgs et al. [18] proposed the Reverse Influence Sampling (RIS) algorithm based on the IC model to find the sources and paths of information propagation by traversing the activated nodes in the network in reverse; however, the use of this algorithm is somewhat limited in the LT model. These heuristic algorithms have limited application in real scenarios because they do not take into account attribute factors such as the emotional attitudes of the nodes, and they are mostly applied in static networks.

### 2.2. Opinion Maximization

Opinion Maximization (OM) is a variant of the IM problem, where the emotional and opinion attributes of users are incorporated. IM-related algorithms enable a broader scope of information dissemination within the IM problem. However, in practical applications, there are both desired and undesired opinions, and the pure dissemination scope and the number of affected nodes cannot meet practical needs. In this regard, Chen et al. [2] proposed the IC-N algorithm based on the independent cascade model, which introduces the propagation of negative opinions and takes into account the community structure and node influence within the network. However, the IC-N algorithm assumes that the influence parameters of users are all the same, which is not in line with the actual situation. Zhu et al. [19] proposed the SRIS algorithm, which is based on the RIS algorithm and takes into account the polarity relationship of nodes in a signed social network, thereby reasonably determining their influence relationships. This allows the positive influence of the seed nodes to spread more widely; however, its application in the LT model is greatly limited. In this regard, He et al. [4] came up with the AOMF algorithm under the LT model to solve the POM problem; however, this algorithm does not take into account the possible existence of silent nodes in the social network or the possible cessation of opinion propagation.

Liu et al. [20] summarized the POM problem, describing it as finding a set of seed users in a multi-round campaign to maximize the overall dissemination of opinions about a target product. They proposed the Cone framework, which estimates users’ opinions about a product through matrix decomposition of their preference data and then selects a set of initial users that can maximize the dissemination of positive opinions based on their opinions and the network’s dissemination model. However, the Cone algorithm does not consider the social relationships or influence among users; it only selects initial users based on their views without considering their social network structure and propagation ability. Subsequently, Li et al. [21] studied the Influence Maximization method of group sentiment and proposed the PUEA algorithm to locate the positive seed nodes.

Although the OM problem has been studied by many parties, there are still limitations, such as the inability to select appropriate seed nodes based on the characteristics of the network structure, which is mostly applied in static networks.

### 2.3. Opinion Dynamics

Opinion dynamics models are essential tools for studying how individual opinions evolve in social networks. People’s opinions evolve dynamically when absorbing information shaped by group influence or authority cues. Dong et al. summarized the process of opinion dynamic fusion into three parts: opinion expression format, fusion rules, and opinion dynamic environment [22]. Based on this, researchers have proposed a series of opinion dynamics models to simulate user opinion dynamics, which can be categorized into discrete and continuous opinion dynamics models according to the method of opinion expression. Discrete opinion dynamics models assume that opinions are limited options, i.e., positive, negative, or neutral. Common models include the voter [23] and majority-rule models [24], which ignore continuous opinion changes. For continuous models, DeGroot et al. [25] proposed the classical DeGroot model, assuming opinions vary continuously between −1 and 1 and evolve via linear combinations of neighbors’ opinions. Friedkin and Johnsen [26] improved DeGroot’s model by anchoring opinions to prejudices, which implicitly introduces “stubborn-like” behavior through predefined self-connections in the network topology. Deffuant et al. [27] proposed the DW model based on this, determining individual interactions in social networks through bounded confidence. Similar to the DW model, Hegselmann and Krause [28] proposed the HK model, stating that nodes update their opinions by considering a weighted average of the opinions of neighboring nodes with finite confidence.

In recent years, researchers have tended to synthesize models and examine user attributes in more detail in the study of dynamic opinions. Ye et al. [11] proposed an EPO algorithm to categorize individual opinions into private and expressed opinions and considered the individual’s self-confidence, sensitivity to interpersonal relationships, etc., in the opinion evolution process. Subsequently, Hou et al. [29] improved this by considering the HK and EPO models together and proposed the MEPO algorithm. Wu et al. [30] proposed the SNHK model, which combines features of the DeGroot and HK models and also considers the strong and weak relationships between individuals.

In all of these opinion dynamics models, there is no limitation on the propagation time of opinions; however, the algorithms assume infinite propagation, resulting in inaccurate results. At the same time, the process of opinion change does not take into account the silent node problem, which is not in line with the silent spiral effect in social psychology [31,32].

## 3. Model

In response to the existing issues in opinion propagation within social networks, such as neglecting silent nodes, one-time seed selection, and overlooking the dynamism of opinion propagation, this section proposes a new solution, the LOMDP framework, to address these challenges. This framework introduces the concept of node expression capacity to identify silent nodes, presents the LOM algorithm for multi-stage seed selection to maximize the propagation of desired opinions, and proposes the LODP model to simulate the dynamic changes in node opinions. This framework provides a more comprehensive and in-depth solution for maximizing the spread of desired opinions.

### 3.1. Subsection

**Figure 1:**
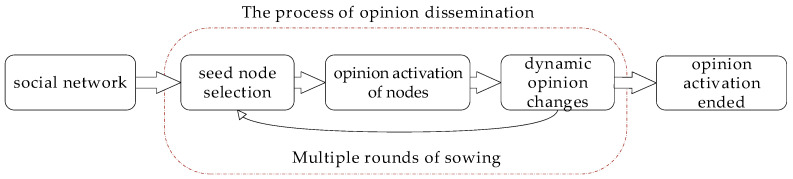
Process of opinion propagation.

Traditionally, the desired opinion maximization problem is divided into three stages: (i) selection of seed nodes and seeding methods, (ii) opinion activation of nodes, and (iii) dynamic changes in node opinions. As shown in Figure 1, appropriate seed nodes are first selected from social networks, and opinions propagate outward layer by layer from these seeds. During the propagation process, opinions dynamically evolve over time. To maximize desired opinions, multi-stage seeding strategies are often adopted until opinion propagation ceases. Our innovations in this process focus on optimizing two key aspects: seed node selection and dynamic opinion evolution modeling. Specifically, we propose the LOM algorithm and the LODP model to enhance previous approaches. Furthermore, we assume that nodes have finite expression capacity and integrate this principle throughout the proposed framework.

We transformed the desired Opinion Maximization problem in a social network into selecting appropriate seed nodes and seeding methods in a graph, G, to maximize desired opinions. We modeled a social network as a directed graph, $G=\left(V,E,W\right)$, where $V=\left\{v_{1},v_{2},\ldots v_{n}\right\}$ are nodes in the graph denoting users in a social network, $E=\left\{e_{1},e_{2},\ldots ,e_{n}\right\}$ are directed connections between nodes denoting connections between users, $W=\left\{w_{ij},1<=i,j<=n,-1<=w_{ij}<=1\right\}$ are weights of edges denoting closeness of users, and w ranges from −1 to 1, denoting that a relationship gradually changes from hostile to close.

### 3.2. Node Expression Capacity

Neumann proposed the spiral of silence theory [31,32] based on the theory of mass communication. According to this theory, when an individual’s view conflicts with the mainstream societal view, they tend to remain silent. We believe that opinion propagation on social networks also follows this communication theory. Therefore, in our research, we applied the spiral of silence theory and propose a concept called node expression capacity to describe the ability of a user to interact with their neighbors. The proposal of this concept takes into account the following factors:

(i) In real social networks, there are inevitably silent nodes. According to cognitive psychology theories [33], every individual has personality traits that influence their behavior. For example, introverts may be reluctant to express their opinions, which may lead them to become silent nodes. In addition, according to social psychology theories [34], people may choose to remain silent due to social pressure from authority or conformity.
(ii) The propagation of an opinion is not endless; it eventually reaches a termination point. One reason for this is that the “heat” of an event gradually diminishes, leading to decreasing user attention over time.
(iii) Silent nodes can impact the selection of seed nodes and the propagation of information. For example, a highly influential sports blogger may choose to remain silent about events in the gaming domain. If the sports blogger is selected as a seed node for propagation based solely on the influence factor, the final desired outcome may be suboptimal, as their silence could hinder the effective spread of the targeted opinion.

Similar to a network environment that alternates between silence and activity [35], we categorize societies into silent societies and active societies. A silent society refers to a society in which individual communication is relatively scarce, and the public may choose not to express their opinions due to social pressure or personal character traits. Conversely, an active society refers to one in which individual communication is frequent, and the public is more inclined to share a wide range of opinions and information.

Different types of societies can impact the spread of desired opinions. For example, in a silent society, the proportion of users expressing extremely positive or negative opinions may be relatively low. At the same time, the public may be more susceptible to the influence of conformity, leading to homogeneous opinions. Therefore, we introduced the variable $D_{0}$ to describe the impact of this social environment.

Based on the abovementioned factors, we propose the concept of node expressive capacity and provide a formal definition for it as the node expression (NE) in Equation (1). NE, an integer usually ranging from 0 to 10, denotes the predicted number of times a user can interact with surrounding nodes in the current environment. The cognitive influence coefficient, φ, is determined by the user’s personality and growth environment. Event heat, H, indicates the degree of attention an event attracts, and the group expression coefficient, $B_{0}$, is designed to consider the bandwagon effect during user expression. This formula reflects the predicted interaction frequency of user nodes with surrounding nodes under the influence of various factors, which can be used to identify silent nodes.

(1) $$NE=\varphi *H*B_{0},$$

(2) $$\varphi =D_{0}*\frac{1}{k}\sum_{j\in N_{in}^{i}}\frac{1}{diff\left(o_{i},o_{j}\right)+1},$$

(i) The cognitive influence coefficient, φ, is defined in Equation (2). The cognitive influence coefficient is determined by a user’s intrinsic and social cognitive characteristics. We chose the personal character as $D_{0}$ to describe users’ intrinsic cognitive characteristic, with $D_{0}\in \;\left[0,1\right]$, where 0 means a user is extremely introverted and 1 means a user is extremely extroverted. Randomly generated values from 0 to 1 are used to represent varying personal characters. Here, j is the in-degree neighbor of node i, $\mathrm{diff}\left(o_{i},o_{j}\right)$ is the opinion difference between node i and node j, and k is the number of in-degree neighbors of node i.

(ii) Event Heat (H). To simulate users’ decreasing attention as an event loses attention, we introduced Newton’s Law of Cooling [36] to model the heat of an event. The calculation of this law is described in Equation (3).

(3) $$\frac{dH}{dt}=\;-\;\alpha \left(H-H_{a}\right),$$

Here, H represents the temperature of an object at time t, signifying the event’s intensity at that moment; dH/dt denotes the rate of change in this intensity; $H_{a}$, the constant ambient temperature, represents the event’s intensity at time t→∞; and α is the cooling coefficient, indicating the rate of intensity decrease.

By solving Equation (3), we obtain Equation (4). Here, C is the coefficient obtained after solving the differential equation.

(4) $$H-H_{a}=C*e^{-\alpha t},$$

We postulate that as t→∞, the thermal intensity of the event decreases to zero, i.e., H=0. At t=0, the thermal intensity of the event is 1, i.e., H=1. Substituting these values into Equation (4), we obtain C=1 and $H_{a}=0$, thus deriving the final equation for the event’s thermal intensity, as shown in Equation (5). Through the assignment of coefficient α, the correlation between time and event heat can be derived.

(5) $$H=e^{-\alpha t},$$

(iii) The group expression coefficient, $B_{0}$, is used to characterize the maximum expressive capacity of most individuals within a specific social environment. $B_{0}$ fluctuates around a base of 10 and can be used to simulate active and silent societies. In an active society, $B_{0}$ may be higher, reflecting more frequent communication and opinion sharing among individuals, whereas in a silent society, $B_{0}$ may be lower, reflecting less communication and opinion expression.

### 3.3. Seed Node Selection Algorithm

In the DOM problem, both desired and undesired opinion nodes emerge during the opinion propagation process. These two types of nodes exert different influences on the process. Therefore, in the first stage of seed node selection, we take into account both the opinion inclination and the influence of the nodes to maximize desired outcomes. Additionally, we identified key characteristics of opinion propagation: (i) opinions initially propagate exponentially; (ii) the growth of activated nodes slows or halts after 5–10 rounds of propagation, resulting in a more dispersed distribution of inactive nodes. Consequently, the rate of opinion spread diminishes over time. Interactions between seed nodes suggest that repetitive selection strategies may lead to suboptimal outcomes with diminishing returns.

Based on these observations, we recognized that the batch selection of all seed nodes or a uniform selection method is inadequate. In response, we introduced the LOM algorithm, which adopts a multi-round seeding approach with diverse strategies to enhance the spread of desired opinions. It carries out iterative seeding, distributing seed nodes across multiple rounds to avoid local optima. By adjusting the seeding strategy according to the network’s evolving dynamics, this algorithm promotes greater adaptability and strategic flexibility in the propagation of desired opinions.

The LOM algorithm starts with the Clauset–Newman–Moore algorithm [37] to partition a directed graph of a social network into different communities. It seeds k seed nodes over l rounds and sows k/l seeds in each round. To filter important communities for a simple computation, it only filters communities with a node count exceeding 50. In the first round of seeding, the number of seed nodes is distributed in proportion to the size of the community and selected using Equation (6) to calculate desired returns. In the subsequent l−1 rounds, Equation (6) is replaced with Equation (7). Algorithm 1 summarizes the whole process of the LOM algorithm.

(6) $$P_{1}\left(i\right)=o_{i}I_{i}+\sum_{\begin{array}{c} j\in N_{i}^{in}\\ {NE}_{j}\neq 0 \end{array}}\left(o_{j}+w_{ji}o_{j}\right),$$

(7) $$P_{2}\left(i\right)=o_{i}+\sum_{\begin{array}{c} j\in N_{i}^{in}\\ {NE}_{j}\neq 0 \end{array}}\left(o_{j}+w_{ji}o_{j}\right),$$

In Equations (6) and (7), $o_{i}$ and $o_{j}$ denote the opinions of node i and node j, respectively, which are obtained from the users’ historical evaluations at the initial stage of the algorithm. $I_{i}$ denotes the influence of node i. Here, we used the PageRank algorithm [13] to estimate the node’s influence. j is the non-silent node among the in-degree nodes of node i, i.e., ${NE}_{j}\neq \;0$. $w_{ji}$ denotes the weights of the directed edges from j to i.

During the initial stage of the dissemination process, we underscored the significance of influential seed nodes, employing Equation (6) to calculate their desired returns. It is our intention that these nodes function as opinion leaders for favorable viewpoints, thus directing public opinion at the outset of dissemination. With the advancement of the propagation process, inactive nodes become more scattered. In Equation (7), by removing the influence factor, the greedy algorithm is poised to deliver greater desired outcomes. Our strategy is designed to shape public opinion by achieving social consensus early in the dissemination process and to continue the spread of desired opinions through the intermediate and later stages, ultimately maximizing the prevalence of desired sentiments.

**Figure 2:**
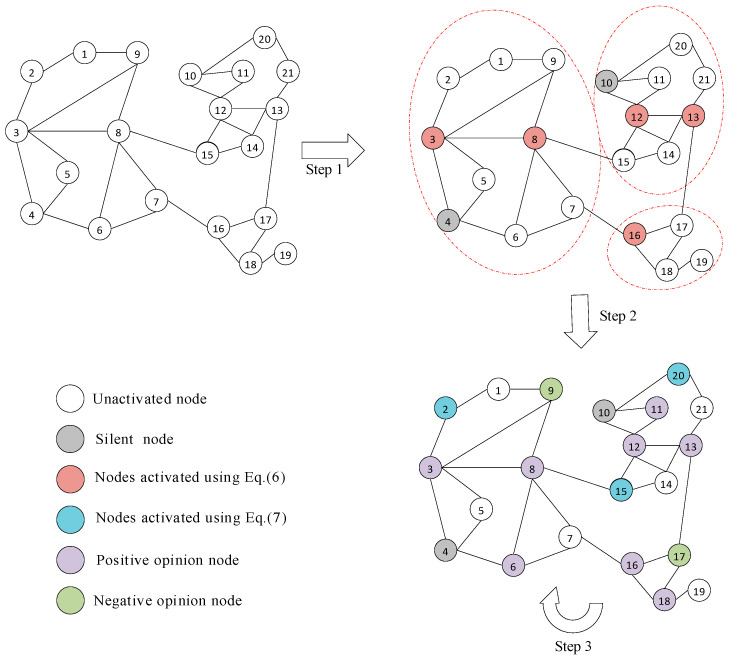
Description of the LOM algorithm.

Figure 2 depicts the whole process of the LOM algorithm. Step 1 involves dividing the community and calculating the seed nodes for the first round of distribution using Equation (6). Step 2 indicates that by the time the propagation process reaches the second round and the seed nodes are calculated using Equation (7), there are already some activated nodes in the social network, including both desired and undesired opinion nodes. Step 3 shows that the propagation process is repeated for seeding at subsequent times until the node budget is exhausted.
**Algorithm 1** LOM Algorithm**Input:** Network G = (V, E, W), the number of seed nodes, k, the number of sowing stages, l, and stage time, T **Output:** list of seed nodes, S **BEGIN** S←∅ **for** r = 1:l, **do**   **if** r == 1, **then**     Divide G into communities: community_dict = {community, nodes_number}     **for** each community, **do**      Seed_counts = k × nodes_number/len(G.nodes)      Seeds <- sort community nodes by Equation (6)      S <- S∪Seeds[:k/l]      **end for**   **end if**   **if** r > 1, **do**     Seeds <- sort G.nodes by Equation (7)     S<- S ∪ Seeds[:k/l]   **end if** **end for** Obtain the seed node set S. **END**

### 3.4. Dynamic Opinion Change Model

In the DOM problem, opinions may change dynamically during the propagation process. To address this, we propose the LODP model. This model is inspired by the classic FJ model [26], which balances a user’s internal beliefs with the influence of their neighbors, and the bounded confidence model [28,29], which assumes that individuals only interact with neighbors who hold similar opinions. By integrating these two approaches, LODP provides a more realistic simulation of opinion dynamics in social networks, accounting for both internal beliefs and selective interaction based on opinion similarity.

The FJ model assumes that an individual’s opinion is influenced by both their intrinsic beliefs and the opinions of their surrounding neighbors, as described by Equation (8). In contrast, the opinion-limited confidence model assumes that individuals only communicate with neighbors who hold similar opinions. Compared to the traditional FJ model, the LODP model demonstrates significant advantages in maximizing desired outcomes, primarily in the following aspects:

(8) $$o_{i}^{t+1}=\frac{s_{i}+\sum_{j\in N_{i}^{in}}w_{ji}o_{j}^{t}}{1+\sum_{j\in N_{i}^{in}}w_{ji}},$$

(i) Introduction of the NE coefficient: The incorporation of a new NE coefficient, representing nodes’ expression capacity, allows for the effective identification and elimination of silent nodes’ influence during the dynamic changes in opinions. This enhancement improves the accuracy of opinion recognition. If NE=0, it indicates a silent node, which will no longer participate in the dissemination of opinions.

(ii) Optimization of the opinion update mechanism: By integrating the principle of limited confidence in opinions, the LODP model more closely mirrors the communication patterns among individuals in reality, thereby enabling a more accurate simulation of the opinion dissemination process in social networks.

Specifically, the opinion changes of node i at moment *t* in the LODP model are described in Equation (9).

(9) $$o_{i}^{t+1}=\frac{s_{i}+\sum_{j\in N_{i}^{in},{NE}_{j}\neq 0}w_{ji}o_{j}^{t}}{1+\sum_{j\in N_{i}^{in},{NE}_{j}\neq 0}w_{ji}},$$

where $o_{i}^{t}$ denotes the opinion of node i at moment t; node j is the activated in-degree neighbor of node i, expressing a non-zero capacity, i.e., ${NE}_{j}\neq 0$, and the difference in opinion with node i is within the confidence level of node i’s opinion; $w_{ji}$ is the weight of the directed edges from node j to node i, where $w_{ji}>0$ denotes a positive relationship, such as trust and friendship, and $w_{ji}<0$ indicates a negative relationship, such as skepticism and hostility; and $s_{i}$ represents the internal opinion of node i, which is shaped by individual factors, such as background, family, and culture. Additionally, the initially received opinions (i.e., the first impression) also play a significant role in the formation of internal opinions. We present the calculation formula for $s_{i}$ as Equation (10).

(10) $$s_{i}=s_{0}+\sum_{j\in N_{i}^{in},{NE}_{j}\neq 0}w_{ji}o_{j},$$

Here, $s_{0}$ denotes the influence of the user’s personal experience, j is the neighboring node of node i when it is first activated, and $o_{j}$ is the opinion of node j. Algorithm 2 describes the process of opinion activation and the dynamic updating of nodes.
**Algorithm 2** Node Activation and Opinion Changes**Input:** Network G=(V, E,W), the number of seed nodes, k, the number of sowing stages, l, and stage time, T **Output:** list of seed nodes, S **BEGIN** **for** t = 1:T, **do**   **for** i in G.nodes, **do**      **if** i is not active, **do**       Initialize in-degree node set V <- ∅       **if** node j is active and $\sum_{j\in V}w_{ji}>\theta$, **do**          node i is activated          update $s_{i}$ by Equation (9)       **end if**      **end if**   **end for**   update opinion i by Equation (8) **end for** **END**

**Figure 3:**
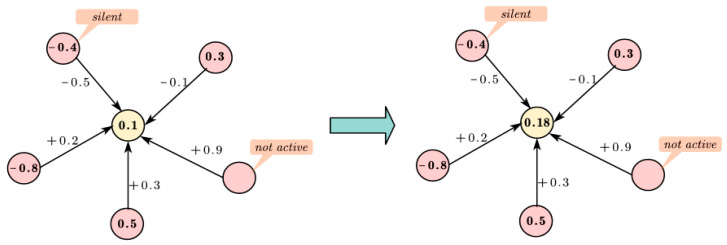
Modeling of opinion dynamics.

The LODP algorithm refines the traditional FJ model by providing a more detailed depiction of opinion dynamics. As illustrated in Figure 3, we considered a central node, i, with an opinion confidence threshold of 0.5 and an internal opinion of 0.1, surrounded by five neighbors, some of which are inactivated or silent. The LODP model computes the subsequent opinion value of node i as [0.1 + (−0.1) × 0.3 + 0.5 × 0.3]/(1 + 0.3 − 0.1) = 0.18. In contrast, the traditional FJ model yields an opinion value of [0.1 + (−0.1) × 0.3 + 0.5 × 0.3 + (−0.8) × 0.2 + (−0.4) × (−0.5)]/(1 + 0.3 − 0.1 + 0.2 − 0.5) = 0.29 for the same node. This comparative analysis demonstrates that the LODP model offers a more granular perspective on node behaviors, leading to a more gradual evolution of opinion values, particularly in the context of the desired Opinion Maximization problem.

## 4. Experiments

To verify the effectiveness of the LOMDP model, we implemented six baseline algorithms using four public datasets. The experimental results show the progressiveness of our method.

Building upon the research in [4], we used the potential opinions of active nodes as one of the evaluation metrics. The potential desired opinion is a commonly used evaluation indicator that can effectively evaluate the effectiveness of a method’s opinion dissemination. The calculation formula for potential desired opinions is provided in Equation (11).

(11) $$\Gamma =\sum_{\upsilon_{i}\in C^{+}}o_{i}+\sum_{\upsilon_{j}\in C^{-}}o_{j\;},$$

Here, $C^{+}$ is the set of activated nodes with a desired opinion, $C^{-}$ is the set of activated nodes with a negative opinion, and $o_{i}$ and $o_{j}$ denote the opinion values of nodes i and j, respectively. By taking into account the overall positive and negative opinions, this formula can comprehensively measure the achievement level of communication goals.

### 4.1. Datasets and Baseline Algorithms

We chose four public datasets for our experiments: Bitcoin Alpha [38], Bitcoin OTC [39], Wiki Vote [40], and Slashdot [41]. All four datasets can be downloaded from the websites http://snap.stanford.edu/data and https://www.aminer.cn/data-sna, both accessed on 10 January 2024. The Bitcoin Alpha and Bitcoin OTC Network datasets collect trust networks among people who use Bitcoin to transact on the Bitcoin Alpha and OTC platforms. The Wiki Vote dataset contains all voting data from Wikipedia’s inception to January 2008. The Slashdot Network dataset contains the friend/foe relationship chains among Slashdot users. The details of the four datasets are presented in Table 1.

We selected five classical algorithms and one improved algorithm that have shown good performance on the Influence Maximization problem. The five classical algorithms are the Degree algorithm [14], the PageRank algorithm [13], the Degree Discount algorithm [16], the IMRank algorithm [42], and the CELF++ algorithm [10]. The improved algorithm is the AOMF algorithm [4].

- Degree algorithm: Calculates node influence based on degree centrality.
- PageRank algorithm: Iteratively computes the importance of web pages based on their linking relationships, with more links indicating higher influence.
- Degree Discount algorithm: Assigns higher influence to nodes with higher degrees but discounts the influence of each connection as the node’s degree increases.
- IMRank algorithm: Models node influence as a probability distribution that reflects both the node’s structural position and its role in information dissemination.
- CELF++ algorithm: Enhances the efficiency of influence calculation by employing an optimized greedy approach that focuses on the incremental gain of influence for each node.
- AOMF algorithm: Estimates a node’s potential positive impact, initiating multiple iterations of linear threshold propagation to simulate influence spread.

### 4.2. Hypotheses and Parameters

To make the experimental results more reasonable, we propose several hypotheses and establish relevant parameters.

In the section on node capacity expression, when calculating the cognitive influence coefficient, φ, the inherent influencing factor, $D_{0}$, of user personality is randomly assigned within the [0, 1] interval. When comparing the performance with the baseline algorithm, we set the group expression coefficient, $B_{0}$, to 10 to simulate a balanced state between active and silent societal states.

In the design of the LOM algorithm, weights are randomly assigned to network-directed edges. Following a 4:1 ratio, there is an 80% probability that the weights fall within the (0, 1] interval and a 20% probability that they fall within the [−1, 0] interval, representing trust relationships between nodes. Since nodes with high influence may hold either desired or undesired opinions, and to ensure fairness in the methodology, only seed nodes with initial opinion values greater than 0 are considered. In the multi-stage seed selection strategy, we set the number of stages from 1 to 5, balancing algorithm complexity and execution efficiency.

Finally, in the LODP dynamic opinion change model, initial opinions $s_{0}$ are generated randomly within the interval [−1, 1]. Considering the uncertainty of user personalities, users’ linear thresholds are randomly generated within the range [0, 1]. Additionally, to enhance the generalizability of the research results, outlier data that significantly deviate from the overall trend are excluded. All experimental data are processed based on the average of 50 simulations. This rigorous experimental design ensures the stability and broad applicability of our research results.

### 4.3. Comparison of the Number of Active Nodes When Considering Expression Capacity

In this section, we investigated the influence of node expression capacity. Previous studies on the DOM problem assumed that interactions between users and neighboring nodes would continue indefinitely without cessation. We demonstrated that this assumption would lead to experimental results that deviate from reality and further examined the impact of this variable on opinion distribution.

**Figure 4:**
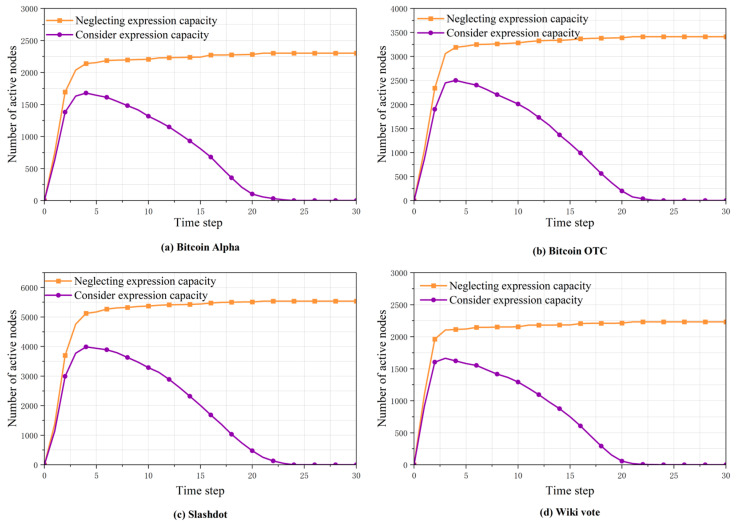
Impact of expression capacity on the number of active nodes.

Figure 4 depicts the number of active nodes in social networks considering expression capacity and ignoring expression capacity, with a population expression coefficient of $B_{0}=10$ and a cooling factor of α=0.2. It can be observed that during opinion propagation, the number of active nodes initially rose rapidly and then gradually decreased, ultimately tending toward zero. When expression capacity was ignored, the number of active nodes remained high, leading to an overestimation of desired opinion propagation compared to actual conditions and making the results less informative.

We further investigated the distribution of propagation in societies with different types of opinions. We divided the opinion values into five categories, as shown in Table 2.

**Figure 5:**
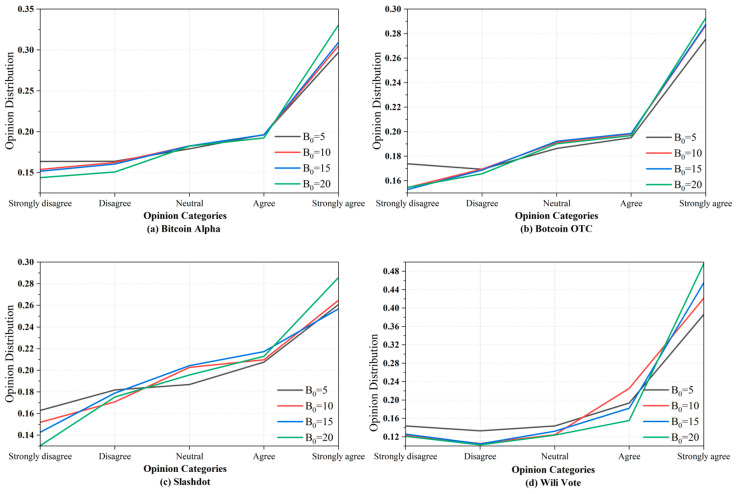
Opinion distribution and expression capacity.

By adjusting the group expression coefficient, $B_{0}$, we can influence users’ expressive capacity, thus simulating silent and active societies. We set $B_{0}$ to 5, 10, 15, and 20 to represent the gradual transition from silent to active social dynamics. By conducting experiments with k=200, we obtained the opinions of all activated nodes and calculated the distribution of different opinion categories. The results are shown in Figure 5, where the horizontal axis represents the types of opinions, and the vertical axis represents the proportion of different types of opinions among the active nodes at the end of propagation. Different colored lines simulate different types of societies.

The experimental results indicate that as the group expression coefficient, $B_{0}$, increases, the distribution curve of opinions becomes steeper, which is more evident in medium-to-large social networks, like (c) and (d). When $B_{0}$ is smaller, for example, $B_{0}=5$, the distribution of opinions appears more uniform, and the curve is smoother. This may suggest that individuals in silent societies are more easily influenced by social pressure and personality, leading them to express moderate opinions and avoid overly extreme viewpoints. In contrast, in active societies, different opinions are more easily spread, and group polarization [5] is more likely to occur, exacerbating the imbalance in the distribution of opinions.

### 4.4. Analysis of Potential Desired Returns

In this section, we conducted three experiments: Alteration I, Alteration II, and the LOMDP method. For the LOMDP method, we calculated the potential desired gains of nodes using Equation (6) during the initial seeding rounds. In subsequent rounds, we transitioned to using Equation (7) for these calculations. This adjustment is based on the observation that, during the initial stages of propagation, the rate of opinion diffusion escalates exponentially. This leads to diminished influence and increased dispersion among non-active nodes in later seeding rounds, making Equation (7) more suitable for assessing desired gains. To substantiate this hypothesis, comparative experiments were undertaken.

In these comparative experiments, the Alteration I algorithm was employed to denote the exclusive use of Equation (6) for calculating potential desired gains, while the Alteration II algorithm was utilized to signify the sole use of Equation (7) for these calculations, with all other variables aligned with the LOMDP method.

**Figure 6:**
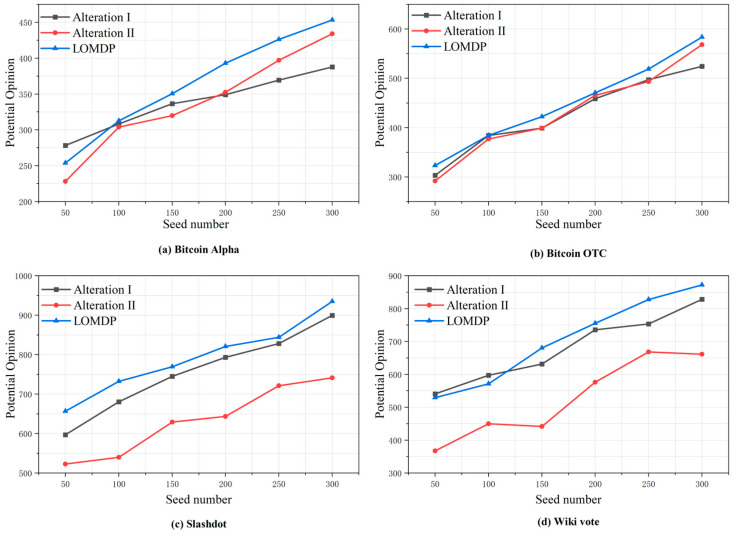
Comparison of the calculation methods for potential desired returns.

Figure 6 illustrates the comparison outcomes. Evidently, dependency on Alteration II alone resulted in notably inferior performance within medium-to-large datasets. In contrast, the Alteration I algorithm demonstrated satisfactory performance in medium-to-large social networks, albeit with slight underperformance in small-scale social networks. Most prominently, the LOMDP method outperformed both Alterations I and II: across datasets, it achieved a performance improvement spectrum of −8.82–16.90% relative to Alteration I (with substantial gains in most scenarios) and 1.09–53.99% compared to Alteration II. Notably, in complex scenarios typified by the Slashdot and Wiki datasets, the improvement frequently exceeded 20%, thereby highlighting the LOMDP method’s efficacy in multi-stage seeding with diverse calculation mechanisms for desired gains.

### 4.5. Comparison of Potential Views

**Figure 7:**
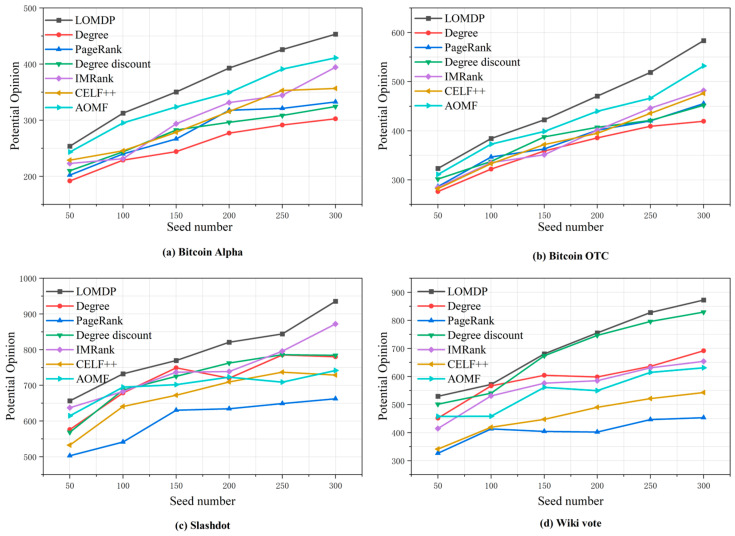
Comparison of potential opinions under different seed quantities.

Based on Equation (11), we calculated the potential desired opinions of various algorithms under different numbers of seeds (ranging from 50 to 300, with a step size of 50). As shown in Figure 7, the LOMDP method demonstrated a significant advantage across the datasets.

(i) In terms of potential desired opinions, in the small datasets, (a) and (b), LOMDP achieved increases of 10.2% and 9.5% versus AOMF at 300 seeds, averaging an 8.7% improvement over AOMF across all seed numbers (50–300). In the large datasets, (c) and (d), it showed enhancements of 7.2% and 5.2% at 300 seeds, maintaining a 6.1% average advantage over the best-performing baselines across all seed scenarios.

(ii) In terms of potential desired opinions, LOMDP demonstrated 100% advantage coverage (4 datasets × 6 seed conditions), outperforming baselines in all seed number settings across both small and large networks. For example, in Alpha, it averaged 23.47% over Degree, 20.12% over PageRank, and 8.97% over AOMF. In Slashdot, it secured average improvements of 17.85% over Degree and 34.02% over PageRank. This consistent dominance across network scales highlights its robust adaptability.

Overall, algorithms like Degree and IMRank consider both the personal opinions and influence of nodes when selecting seed nodes, but they do not fully take into account the opinions of neighboring nodes. In contrast, although the AOMF algorithm considers the potential impact of neighboring nodes, it ignores the influence of the nodes themselves. This may lead to the selection of seed nodes that have desired opinions but limited influence in larger social networks, thereby hindering the rapid spread of desired opinions.

In contrast, the LOMDP method takes a holistic approach and considers the opinions of nodes, their influence, and the opinions of their neighboring nodes when selecting seed nodes. At the same time, it allocates seed nodes based on the size of the community, ensuring the rapid spread of desired opinions to form a group effect and reach a social consensus. Therefore, our algorithm can generate greater benefits from desired opinions and demonstrate stable and excellent performance across different social networks.

### 4.6. Analysis of Potential Opinions and Propagation Time

**Figure 8:**
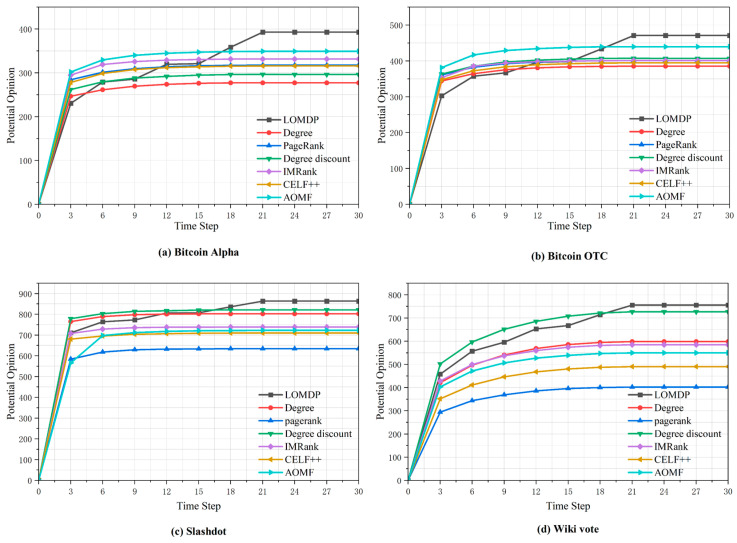
Potential opinions and propagation time.

Figure 8 illustrates the relationship between potential opinions and propagation time for nodes with a seed count of 200. The experimental results confirm the significant characteristics of viewpoint dissemination: (i) exponential growth in the initial stage; (ii) after 5–10 rounds, the speed slows down, and inactive seeds become difficult to activate using the original strategy.

In the four datasets, our algorithm improved by 12.6%, 7.1%, 5.2%, and 4.0% compared to the optimal baseline, demonstrating stable and robust performance across datasets. Although, in the early stages of propagation, the use of multiple rounds of propagation resulted in a slower propagation speed than the baseline algorithm, it maintained sustained growth in performance and ultimately exceeded the baseline algorithm. The other algorithms showed different effects across different network structures. For example, the Degree algorithm performed well in networks with high centrality but poorly in Bitcoin OTC datasets with dispersed nodes. The AOMF algorithm performed stably in small networks, but its effectiveness was limited in large networks (datasets (c) and (d)).

The multi-round seeding of LOMDP solves the problem of budget waste by activating all seeds at once. Unlike AOMF, which only selects subsequent seeds from a predefined set of candidate seeds, the LOM algorithm selects seeds from the entire inactive node network, resulting in LOMDP outperforming AOMF by 12.6%, 7.1%, 19.5%, and 37.5% in the four social networks, respectively.

## 5. Conclusions

This study addresses the problem of maximizing desired opinions in social networks by proposing the LOMDP framework. The proposed method identifies silent nodes by quantifying node expression capacity, employs the LOM algorithm for multi-stage seed selection optimization, and constructs the LODP dynamic propagation model to simulate opinion evolution. The experimental results demonstrate that the algorithm proposed in this paper significantly outperforms traditional algorithms on multiple datasets, achieving over a 10% improvement in potential desired opinion gain while effectively suppressing extreme polarization and ensuring stable propagation performance across diverse networks. Theoretically, the node expression capacity mechanism and entropy-driven propagation optimization provide novel approaches for modeling information uncertainty. Practically, this framework offers efficient tools for scenarios such as public opinion guidance and precision marketing, presenting a new perspective for opinion management in social networks.

## Figures and Tables

**Table 1:** Details of the four datasets.

Dataset	Nodes	Links	Avg. Degree
Bitcoin Alpha	3783	34,186	18.07
Bitcoin OTC	5881	35,592	12.10
Wiki Vote	7115	103,689	29.15
Slashdot	13,182	516,575	78.37

**Table 2:** Opinion categories.

Opinion Values	Opinion Categories
−1≤o<−0.6	Strongly disagree
−0.6≤o<−0.2	Disagree
−0.2≤o<0.2	Neutral
0.2≤o<0.6	Agree
0.6≤o≤1	Strongly agree

## Data Availability

The experimental data in this paper can be provided upon request via email to researchers interested in this study.

## Abbreviations

The following abbreviations are used in this manuscript:

LOMDP	Limited Opinion Maximization with Dynamic Propagation Optimization
POM	Positive Opinion Maximization
DOM	Desired Opinion Maximization
LOM	Limited Opinion Maximization
LODP	Limited Opinion Dynamic Propagation Optimization Model
IM	Influence Maximization
OM	Opinion Maximization
CELF	Cost-Effective Lazy Forward
CELF++	Cost-Effective Lazy Forward Selection ++
SAIM	Social Action-Based Influence Maximization Model
MIA	Memetic Influence Algorithm
RIS	Randomized Incremental Search
IC	Independent Cascade Model
LT	Linear Threshold Model
IC-N	Independent Cascade-Negative model
SRIS	Signed Reverse Influence Sampling
AOMF	Activated Opinion Maximization Framework
PUEA	Potential User Discovery based on Emotion Aggregation
FJ	Friedkin–Johnsen Model
DW	Deffuant–Weisbuch Model
HK	Hegselmann–Krause Model
EPO	Expressed Private Opinion
MEPO	Modified Expressed Private Opinion
SNHK	Social Network Hegselmann–Krause
NE	Node Expression
